# Association of exclusive smokeless tobacco consumption with hypertension in an adult male rural population of India

**DOI:** 10.1186/1617-9625-5-15

**Published:** 2009-11-24

**Authors:** Ambarish Pandey, Nivedita Patni, Sasmit Sarangi, Mansher Singh, Kartavya Sharma, Ananth K Vellimana, Somdutta Patra

**Affiliations:** 1 Centre for Community Medicine, All India Institute of Medical Sciences, New Delhi, 110029, India

## Abstract

**Introduction:**

Tobacco consumption is a major source of mortality and morbidity in India . Prevalence of smokeless tobacco (ST) consumption in India is around 20%. Studies have shown increased prevalence of cardiovascular disease risk factors and an increased incidence of adverse cardiovascular events among the ST consumers. This is a cross-sectional study done to look into the association of exclusive smokeless tobacco consumption with hypertension, in an adult male rural population of north India.

**Methods:**

All male residents of a village in north India above 15 years of age, who did not have any acute or chronic morbidity were included after taking an informed consent. Subjects were interviewed regarding their demographic profile, socioeconomic status and tobacco consuming habits. Current smokeless tobacco user was defined as one who has ever consumed tobacco orally in past 1 month. Blood pressure of the subjects was also recorded. Cut offs used for systolic and diastolic hypertension were 140 mm hg and 90 mm Hg respectively.

**Results:**

443 subjects were included in the study. Prevalence of exclusive ST users was 21% while 19.4% consumed both forms and 26.6% did not take any form of tobacco. Mean systolic and diastolic BP were significantly higher in exclusive ST users(systolic BP=139.2+17.4,diastolic BP = 86.8+11.5)as compared to the non users(systolic BP= 135.7+18.8 , diastolic BP= 82.6 +11.5; p value < 0.05). The prevalence of diastolic hypertension was significantly higher in exclusive ST users as compared to non users ( 40.9%, 22.9% ;p value = 0.01) . The OR for diastolic hypertension in male ST users was 2.3( 95% C.I. = 1.3-4.3). Prevalence of systolic hypertension was higher in exclusive ST users too though this was not statistically significant (43%,36.4%;p value = 0.39.).

**Conclusion:**

ST consumption is associated with increased prevalence of high BP in the adult male rural population.This is an indicator of increased predisposition to major adverse cardiac events later in their life time. Prevention of ST consumption could be an important intervention in preventing the ongoing upswing in prevalence of chronic heart disease.

## Introduction

Tobacco consumption is a major source of mortality and morbidity in India. According to estimates there are approximately 5 million deaths due to tobacco consumption annually which is expected to reach 10 million by 2025. Currently over 20% of worldwide tobacco related mortality occurs in India [1,2].

In developing countries like India, tobacco consumption is mainly done in two forms: smoked tobacco products and smokeless tobacco. Most commonly used smokeless tobacco products include - tobacco pan masala, tobacco with lime, tobacco with pan and betel quid [3]. Prevalence of smokeless tobacco consumption in India is 20%. It is significantly higher in males than in females (28% in males and 12% in females) and in rural population as compared to urban population [4]. Easy affordability, lesser cost and misconceptions regarding its useful health effects are important contributory factors for increased smokeless tobacco consumption.

Association of smokeless tobacco consumption with occurrence of adverse cardiovascular events like myocardial infarction, stroke, and ischemic heart disease has been studied in detail in western population. Results from these studies paint a mixed picture with some showing increased incidence of these adverse events [5-8] while others showing no such association [9-12]. Similarly, contradictory results have been seen in studies evaluating increased risk factors for cardiovascular diseases in smokeless tobacco consuming population [13-17]. In India, limited studies have shown that tobacco chewing is associated with increased prevalence of cardiovascular risk factors like dyslipidemia, hypertension, and ECG abnormalities as compared to non tobacco users [16-18].

This is a cross-sectional study done to look into the association of exclusive smokeless tobacco consumption with hypertension, a well known risk factor for development of cardiovascular disease, in an adult male rural population of north India.

## Materials and methods

A community based cross sectional study was carried out in the village Piayala of Faridabad district in Haryana state in north India over a period of one month. The site of study was chosen by random selection. All male residents of the village above 15 years of age were included after taking an informed consent. Exclusion criteria for the subjects included presence of any self reported acute illness, diagnosed cardiac, renal or hepatic disease, any current treatment for cardiac or blood pressure related morbidities and history of heavy alcohol or recreational drug use. Convenience sampling was used by approaching the subjects for interviewing in the morning before they left for their work.

A peer reviewed, pretested proforma was used to interview the study subjects. The proforma contained questions pertaining to the demographic profile, socioeconomic characteristics, and tobacco consumption habits. For the study, smokeless tobacco was defined as form of tobacco consumed orally and not smoked, and included moist oral snuff, chewing tobacco and tobacco used with betel quid, areca nut, Pan Masala. Current tobacco user was defined as one who has ever consumed tobacco in any form in past one month. Current smokeless tobacco user was defined as one who has ever consumed tobacco orally in past 1 month. A never user was defined as one who has never consumed tobacco in any form. The subjects were interviewed in Hindi on a one to one basis. We also measured the blood pressure of the patient using an electronic BP measuring instrument (OMERON M4) in the right arm standing position. The B.P was recorded on 2 different occasions at 5 minute intervals and the average of the two values was calculated. Systolic hypertension was defined as systolic blood pressure more than 140 mm Hg and diastolic hypertension was defined as diastolic blood pressure more than 90 mm Hg.

Data was entered using Microsoft excel spread sheet and analyzed using SPSS 17.00 version statistical analysis software. Age adjusted Odds ratio was calculated to look into association between the desired variables using logistic regression models. Chi square test was used to test the association between categorized variables and independent T test was used to compare means of continuous variables.

## Results

The male population of the village with age greater than 15 yrs was 554 out of which 36 (6.5%) were not available at the time of interview. 44 (7.9%) were excluded due to lack of consent and 31(5.6%) were excluded due to presence of either acute illness (n = 7) or a history of pre existing cardiovascular disease (n = 9) or current blood pressure medication (n = 11) or chronic alcoholism (n = 4). Finally, 443 subjects were included in the study. Prevalence of consumption of different forms of tobacco (smoked vs. smokeless) is shown in Table 1. The demographic profiles exclusive smokeless tobacco users and non users of tobacco are shown in Table 2. Multivariate logistic regression analysis showed a statistically significant correlation between exclusive smokeless tobacco consumption and less than 5 years of education among the subjects (Table 3). Also odds of exclusive smokeless tobacco use were significantly higher in unskilled or semiskilled workers like farmers, factory workers and daily laborers (Table 3). Prevalence of risk factors for hypertension, mean values of B.P and the prevalence of systolic and diastolic hypertension in the population are shown in Table 4. No statistically significant difference was seen in prevalence of any risk factor among exclusive smokeless tobacco user and non user population. Mean systolic and diastolic blood pressure were higher in exclusive smokeless tobacco user population as compared to the non users. Prevalence of systolic hypertension was higher in exclusive smokeless tobacco consumers as compared to non users. However this difference was not statistically significant (p value = 0.39). The Odds Ratio adjusted to age, BMI, exercise and family history of hypertension for systolic Hypertension among the exclusive smokeless tobacco users was found to be 1.4(95% C.I. = 0.8-2.7). The prevalence of diastolic hypertension was found to be significantly higher in exclusive smokeless tobacco user male population as compared to non users of tobacco(p value = 0.01). The adjusted odds ratio for diastolic hypertension in male smokeless tobacco users was found to be 2.7(95% C.I. = 1.4-4.9)

**Table 1:** Prevalence of consumption of different forms of tobacco in rural male population

Mode of tobacco consumption	% Distribution (n = 443)
Non user of tobacco	26.6%(n = 118)

Exclusive Smokeless tobacco user	21% (n = 93)

Exclusive smoker of tobacco	33% (n = 146)

Both smoker and smokeless tobacco user	19.4%(n = 86)

**Table 2:** Demographic details of exclusive smokeless tobacco users and non users of tobacco

Baseline characteristics	Exclusive smokeless tobacco user(n = 93)	Non users of tobacco (n = 118)
Mean Age	35.4 ± 11.2 yrs	36.3 ± 13.1 yrs

age group distribution	<20: 14% 20-40: 60.2% 40-60: 16.2% >60: 9.6%	<20: 17.7% 21-40: 55.9% 41-60: 15.2% >60:16.1%

Marital Status	Married: 77.4% Unmarried:22.6%	Married: 72.2% Unmarried: 27.8%

Literacy	<5 yrs: 62.3% 6 yrs or more: 38.7%	<5 yrs: 52.4% 6 yrs or more: 48.6%

Occupation	Farmer: 21.1% Factory worker: 20.2% Laborers: 25.3% Students: 5.2% Other: 25.1%	Farmer: 23.2% Factory worker: 15.5% Laborers: 11.4% Students: 10.3% Others: 36.3%

**Table 3:** Logistic regression analysis of exclusive smokeless tobacco consumption and demographic characteristics

Characteristic	Chi square	Odds ratio for exclusive ST consumption	P value
Age: <45 (RC) >45	0.034	0.91(0.36-2.28)	0.85

Marital status Unmarried(RC) Married	0.797	1.33(0.71-2.50)	0.37

Literacy: < 5 yrs of Education(RC) >5 yrs of Education	6.85	0.48(.27-0.83)	0.009

Occupation: Unskilled/Semiskilled (RC) Others	6.01	0.49(0.27-.87)	0.014

P value < 0.05 is considered significant. RC: reference category; Unskilled and semi skilled workers include farmers, daily laborers, and factory workers

**Table 4:** Comparison of blood pressure and other relevant parameters among exclusive smokeless tobacco users and nonusers

	Exclusive ST user (N = 93)	Non user of tobacco (N = 118)	O.R.* (95%C.I)	P value
Mean body weight (Kg)	51.8 ± 10.4	53.2 ± 12.5	-	0.386

Family history of HTN	5.4% (n = 5)	6.77% (n = 8)	-	0.779

regular exercise	12.9% (n = 12)	11.86% (n = 14)	-	0.835

Mean systolic B.P(mmHg)	139.2 ± 17.4	135.7 ± 18.8	-	0.16

Mean diastolic B.P(mmHg)	86.8 ± 11.5	82.6 ± 11.5	-	0.01

Systolic HTN prevalence	43% (n = 40)	36.4% (n = 43)	1.4 (0.8-2.7)	0.39

Diastolic HTN prevalence	40.9% (n = 38)	22.9% (n = 27)	2.7 (1.4-4.9)	0.0018

Mean systolic and diastolic blood pressures in exclusive smokeless tobacco users and non users of tobacco along with prevalence of systolic, diastolic hypertension, and other associated variables in the population.

## Discussion

Smokeless tobacco consumption is associated with increased prevalence of high blood pressure in the adult male rural population. This finding is similar to some previous studies done in Indian [16-18] as well as western population [19-24]. Significantly higher prevalence of diastolic blood pressure in the smokeless tobacco users as compared to non-users corroborates with findings in previous studies [16,19]. However many studies have failed to show an association between smokeless tobacco use and hypertension [14,25-28]. This could be due to difference in subject population included in these studies. Also, differences in the study designs (cross sectional vs. prospective) and in adjustment for various confounding factors could be contributing to the variability in the results. Other possible reasons for this population based difference in the effect of smokeless tobacco on blood pressure could be due to difference in the composition of the smokeless tobacco used as well as difference in predisposition to HTN in populations of different origins. Availability of Data for Indian population, in this regard, is very limited. Studies have shown that smokeless tobacco acutely increases blood pressure and heart rate similar to tobacco smoking [20]. Acute Nicotine exposure from smoking cigarettes is a well known factor for causing adverse cardiovascular outcomes [29]. Evidence suggests that chewing tobacco leads to blood nicotine levels similar to that seen in smoking. Moreover, due to prolonged absorption, high levels of nicotine are achieved for longer durations of time. The sympathicoadrenal activating properties of nicotine and high sodium content of oral tobacco preparations could be the main contributing factors for high B.P in tobacco chewers [29]. This high content of sodium could be more contributory to the diastolic blood pressure than systolic as shown in our study results. Further studies are warranted to gain insight and understanding of this matter.

## Limitations

This study included the study population of one village only, thus leading to some degree of selection bias. Differences in dietary habits of the subject, like amount of salt intake, have not been taken into account which could be a potential confounding factor. Also, recall bias could have been there in self reporting of illnesses by the subjects.

## Conclusion

An increased prevalence of high blood pressure is seen amongst asymptomatic males who are exclusive smokeless tobacco users. This is an indicator of increased predisposition to major adverse cardiac events later in their life time. Prevention of Smokeless tobacco consumption could be an important intervention in preventing the ongoing upswing in prevalence of chronic heart disease that is threatening to engulf every region of the world.

## Abbreviations

ST: smokeless tobacco; BP: Blood pressure.

## Competing interests

The authors declare that they have no competing interests.

## Authors' contributions

This study was done as a part of curriculum for final year of medical school by students of 2003 MBBS batch of All India Institute of Medical Sciences. AP and NP contributed equally to the study. They were involved in the study designing, literature review, data collection, and analysis and manuscript preparation. MS, KS, AKV were involved in data collection and data analysis part of the study. SS was involved in data analysis and manuscript writing. SP was involved in supervision of the study and helped with designing the study. All the authors have read and approved the final manuscript.
